# Defining cricket batting expertise from the perspective of elite coaches

**DOI:** 10.1371/journal.pone.0234802

**Published:** 2020-06-15

**Authors:** Jonathan Douglas Connor, Ian Renshaw, Damian Farrow

**Affiliations:** 1 Department of Sport and Exercise Science, James Cook University, Townsville, Australia; 2 Institute for Health and Sport, Victoria University, Melbourne, Australia; 3 School of Exercise and Nutrition Sciences, Queensland University of Technology, Brisbane, Australia; 4 Australian Institute of Sport, Canberra, Australia; Manchester Metropolitan University - Cheshire Campus, UNITED KINGDOM

## Abstract

Traditionally in sporting tasks, expertise has been thought of as the attainment of near flawless technical abilities. While contemporary views have become more holistic in nature, in certain sporting domains it is still not clear what exactly encapsulates expertise. This study sought to further understand the crucial and defining characteristics of cricket batting; a complex and difficult perceptual-motor skill with minimal error tolerance and severe time constraints. Eight elite cricket batting coaches, who themselves were former international or state level batsmen, were interviewed to identify characteristics of cricket batting expertise. From this, a conceptual model was developed in relation to an expert within their performance environment. This model highlights several key factors experts possess beyond just technical proficiency, such as self-awareness of their technical and tactical strengths in relation to the situation of the game; self-regulatory behaviours to problem solve performance challenges in-game; and psychological strategies such as between-ball routines to manage cognitions and emotions. The conceptual model of batting expertise described in this paper is designed to introduce an order to how these various skills, possessed by an expert batter, interact within the performance environment to interpret expert performance.

## Introduction

Successfully intercepting a fast moving object requires individuals to develop superior skills that provide an ‘expert advantage’. Previous research across fast ball sports has highlighted that experts develop coordinative, cognitive, perceptual, and psychological advantages to assist in circumventing the extreme temporal and spatial demands associated with interceptive timing tasks [1, 2]. Cricket batting is one such task vehicle commonly utilised by researchers to explore expert advantages. For example, Regan [3] highlighted that at the highest level, batsmen must maintain spatial errors of less than 5cm, and temporal errors of less than 2 to 3ms for deliveries travelling at 160km^-^h. Given the minimal error tolerance permitted in order to be successful, cricket batting is an ideal task vehicle to better understand the complex nature of expertise.

Research into cricket batting expertise has had a strong focus on the individual and their skill capabilities. For example, a cricket batter’s technical skills [4, 5], perceptual capabilities (with particular reference to anticipation) [6, 7] and psychological traits [8] have all been independently investigated. Possessing superior technical abilities, such as earlier initiation of movements, are thought to allow for better execution when striking a cricket ball (i.e., spatial accuracy), and has been examined empirically by manipulating bat width to compare the performance of different skill level batters [9]. Similarly, mental skills such as the ability to manage internal pressures (e.g. anxiety, arousal), are strategies reportedly utilised by skilled cricket batters to achieve more consistent performances [10]. While these are all undoubtedly critical factors of expertise, the importance of providing context when examining skills has been an area of concern for researchers.

Exploring the different characteristics of expertise has required researchers to develop resourceful and inventive methodologies. Video simulations using occlusion techniques [6, 11, 12], pattern recall experiments [13] and laboratory-based experiments [14] are all examples of methods that have advanced our understanding of the various skills possessed by experts. However, an issue of ecological validity has been raised when using these methodologies [15, 16]. Mann and colleagues [17] reported how action specificity is an integral component of an expert cricket batter’s anticipatory advantage; their findings highlighting the importance of utilising tasks where the perception-action couplings are preserved in order to better understand expertise [18, 19]. One unresolved issue stemming from methodologies that are lacking in ecological validity is exactly how contextual information is utilised in a performance environment. Specifically, how does an expert’s own individual constraints (i.e., perceived capabilities such as technical and tactical strengths and weaknesses; emotional states; intentions; fatigue level), in interaction with the dynamic environmental (i.e., pitch and atmospheric conditions) and task constraints (i.e., the current state of the game; position of fielders), influence their decision-making behaviour?

Capturing expertise allows for an understanding of how the dynamic interactions of constraints [20], such as the exemplars described above, influence the emergence of skilled behaviours. Key information sources guide performer’s actions, and unrepresentative experimental designs that exclude these key information sources have shown that experts often lose their performance advantage [21]. Araújo and Davids [22] extended upon the work of Gibson [23] and his original concept of ‘knowledge of’ and ‘knowledge about’ the environment, in order to better understand how interacting constraints and specifying informational sources guide expert behaviours [24]. In this instance, ‘knowledge of’ the environment refers to the individual’s ability to perceive the performance environment in relation to themselves and their own action capabilities. In contrast, ‘knowledge about’ involves indirect perception to capture what information sources mean. How these different forms of knowledge manifest in experts during performance is yet to be fully explored.

Attuning to more specifying information allows the expert to calibrate their actions to exploit available affordances, and subsequently achieve their performance goals [25]. However, an interesting novelty in sporting tasks is the constantly evolving performance goals and environments, which experts must navigate. For example, the immediate goals of a cricket batter at the beginning of a game is likely to be different than half way through the game; likewise the physical performance environment and opposition strategies would change also. Therefore, exploring the ways in which experts attune to specifying information promotes viewing performance as a series of events that are nested within one another, rather than a series of unrelated events [26]. As opposed to a single isolated trial, emergent behaviours can be influenced by factors or situations that happened in previous events (i.e. games, rallies, deliveries, etc.). In his interview with a former expert batter and international level coach, Renshaw [27] reinforced this idea suggesting that performance is not solely about one key event (e.g. a single ball that results in the dismissal of the batter), but instead is a culmination of critical nested and connected events leading up to that particular outcome.

Given the challenges of capturing the true nature of expertise through laboratory-based studies, researchers have begun to explore other methodologies. One such approach has been to utilise coaches. As an untapped knowledge source, they possess a unique wealth of knowledge regarding the specific multidimensional nature of expertise in their sport [28]. Professional and experienced coaches have developed, and continually refine their knowledge, through current and previous experiences, via mentors or peers, trial and error and formalised coaching courses [29]. Utilising this experiential knowledge to explore further the understanding of expert development is therefore an alternative and complementary methodology building upon previous empirical work [30]. The aim of this study was therefore to utilise the knowledge of expert cricket batting coaches to explore the key factors that characterize cricket batting expertise. Given the complex nature of expertise in sport, it is logical to draw upon this experiential knowledge to assist with our understanding. For example, Renshaw [27], identified crucial concepts within the game based on the coach’s unique and extensive experiences. While certain limitations surrounding the use of experiential knowledge to guide empirical research have been noted [31], it is argued that they still provide an ideal platform to investigate the dynamic and complex nature of cricket batting expertise.

## Material and methods

### Participants

Approval for this study was obtained through Victoria University Ethics Committee and all participants provided written informed consent. The participants were eight expert high performance Australian coaches. In order to ensure a well-rounded approach to analysing cricket batting expertise from a group of individuals with different experiences, coaches were required to meet all of the following criteria to be included in the study had; (1) played at a state or international level as a batsmen; (2) coached a state or international team, or be a specialist batting coach in these teams, (3) 5 or more years of coaching experience and (4) a level 3 Australian cricket coaching qualification. All coaches had previously coached more than one state or international team, while the average duration of their coaching at their highest level was 2.8 years ± 2.25 (range; 1.5 years– 8 years). Participants were recruited progressively until data saturation was reached, which was consistent with previous studies’ sample size of expert coaches within a sport [28, 30].

### Data collection

A qualitative descriptive design methodology incorporating one-on-one, in-depth, semi-structured interview techniques was used for the purpose of this study. While structured interviews require all participants to be asked the same questions, in the same order, and formulated ahead of time, a semi-structured approach allows for more flexibility when asking questions [32]. Therefore, a semi-structured approached was employed to better explore the unique individual perspective of each coach with respect to their knowledge and beliefs on batting expertise. In-depth interviews and open-ended questions are common interview techniques and suggested as ideal for eliciting expertise from expert persons [33, 34].

Three investigators were primarily involved with the interviewing process. A series of pilot interviews were conducted with numerous coaches of various coaching levels. This was undertaken, firstly, to ensure the pre-conceived interview questions were appropriate; secondly, to narrow down the ideal coaching level of potential candidates; and thirdly, to provide the first author the opportunity to develop his interview skills specific to this study through discussion and reflection with the second and third author. The first author conducted all eight interviews with expert high performance coaches. Interviews were conducted wherever each coach felt most comfortable, which included an office, empty cricket stands or a coffee shop. Each interview started with a general overview and information about the study. Following this, coaches were asked questions relating to their demographic and coaching experiences (e.g., duration, highest level). The design of the questions were based on Spradley [35] and Cotè and colleagues [33] three categories of open-ended questions; descriptive, structural and contrast questions. Descriptive questions are those that allow the coach to describe their activities and identify what they perceive as being important. Examples of the pre-conceived descriptive questions include “could you describe what batting skill means to you?” or “so what makes for a skilful batter?” or “what are the keys to batting successfully?” Structural questions are those that allow the coach to explain these concepts deeper and for the investigator to understand how this information is organised. Examples of the pre-conceived structural questions include “you talked about the necessity of a (e.g. good technique), could you tell me what a (e.g. good technique) is and what it’s made of?” or “you mentioned experts have a good understanding of where things should be, could you explain what you mean by that?” Finally, contrast questions are those that clarify and distinguish between concepts or ideas coaches describe. Examples of these pre-conceived questions include “what do you believe to be the differences between those who make it beyond a professional representative level and those who don’t?” or “what separates those at a high level, from those playing high level grade cricket?” After each interview, both the first and second author created initial codes, focused codes, and categories after reading through the transcript separately.

### Data analysis

All interviews were transcribed verbatim and sentence-by-sentence open coding was performed by the lead author and co-author to examine any themes identified from coach’s direct quotations [36]. Both authors reviewed their initial and then focused coding, which after identifying consistent concepts emerging, lead to the creation of categories and sub-categories that captured the fundamental meaning or concepts being described by coaches. Similar to Weissensteiner and colleagues [30], a hierarchical method was utilised to conceptualise the higher order (categories) and lower order (sub)categories, and the relationship that existed between them to form a conceptual model. Direct quotations were then used to support each author’s interpretation of the coach’s opinion regarding the ideas being discussed, by placing each quotation in the category or sub-category deemed most appropriate.

A constant comparative process was also used throughout this process. Firstly, the second author was involved after each interview by reading through the transcript and recording their own ideas and concepts. Group meetings (between the first and second author) were conducted to discuss interview transcripts, while key quotes were shared between all authors to elucidate perceived reoccurring themes [37]. Secondly, the first author used a journal to detail key aspects of each interview including concepts, ideas and questions from the responses of the participants. The interviews with coaches continued until theoretical saturation had been generated, whereby no new ideas or concepts emerged. The average duration of interviews were 38 minutes.

## Results

**Fig 1:**
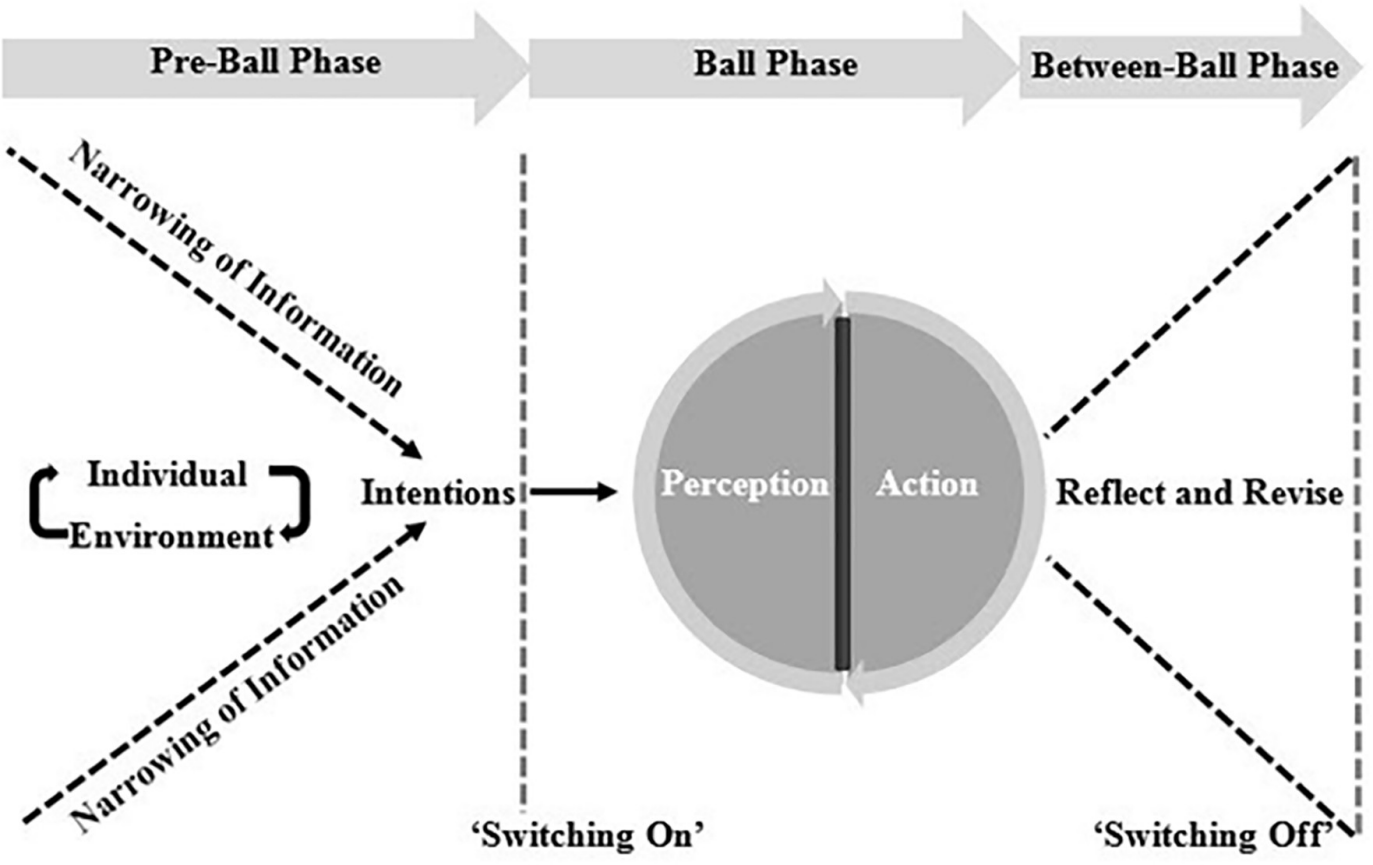
Conceptual model of the process in which an expert batter’s attunement to crucial information shapes their intentions, and precedes both an evaluative and ‘switch off’ phase.

Results emerged from the data collection, and analysis, of interviews comprising 8 expert level batting coaches relating to the factors/processes that they believed underpin cricket batting expertise (Table 1). The critical underpinning processes that constitute expert batting are shown in Fig 1. To set the key processes in context and to simplify the discussion, the findings are organised via a temporal timeline that consists of three phases, including the pre-ball phase, ball phase and between-ball phase. The following section, firstly, describes the model that emerged from the author’s interpretation of the participant’s views, and secondly, explores each phase of the model with direct supporting quotes from the participants.

**Table 1:** Coaching and playing experience of high performance coaches who participated in this study.

Participant	Highest coaching level	Highest playing level
**IC IB**^**1**^	International	International
**IC IB**^**2**^	International	International
**IC IB**^**3**^	International	International
**IC SB**^**1**^	International	State
**SC IB**^**1**^	State	International
**SC IB**^**2**^	State	International
**SC IB**^**3**^	State	International
**SC IB**^**4**^	State	International
**SC SB**^**1**^	State	State

Individual data on coaching duration is excluded due to being easily identifiable (IC = International level coach; SC = State level coach; IB = International level batter; SB = State level batter).

The pre-ball phase begins at a nominal point in time when the expert batter begins to focus attention on an upcoming game. This begins with a generalized search for information about factors that might influence upcoming performance, such as environmental conditions or fixture lists. As the first ball of the batter’s innings draws closer, the search for information becomes more specific. For example, as the batter walks out to bat, information such as atmospheric and pitch conditions, situation of the game and opposition field settings are all relevant in shaping the intentions of the expert batter. Most importantly, these intentions shaped by the batter are dependent on the batter’s knowledge of their own batting capabilities.

The ball-phase, beginning as the bowler enters the run-up stage of their bowling delivery, includes the direct perception and action process that occur as the bowler delivers the ball and the batter is required to coordinate a motor action. Expert batters underpin their action, based on both their intentions, established in the pre-ball phase, and the perception of key information regarding the opposition bowler’s movement. In this sense, both the contextual information about the game and the key perceptual information (i.e. opposition kinematics and ball trajectory) are responsible for the subsequent coordinative action of the batter.

Finally, the between-ball phase occurs immediately after the batter has executed an action, and concludes as the bowler begins their run-up again for the following delivery. Batters begin by reflecting on the previous delivery and the shot they played in response. This is followed by a ‘switching off’ of attending to task relevant information, both cognitively and behaviourally. Finally, expert batters described a ‘switching (back) on’ point whereby batters would begin a set of consistent, routine movements to help focus their attention on the upcoming delivery.

These findings indicated that expert batting is based on a continuous cycle of updating ‘knowledge of’ the individual batter-environment interaction, as a means to achieve their overarching goal of ‘controlling the game’. This ‘knowledge of’ the environment enables batters to develop batting plans to frame intentions in advance of each delivery. Having clear intentions enabled batters to attune to the specifying information, provided by bowlers in their run-ups, that manifested itself as batters getting into a ‘rhythm’ with the bowler’s approach/ run-up.

**Fig 2:**
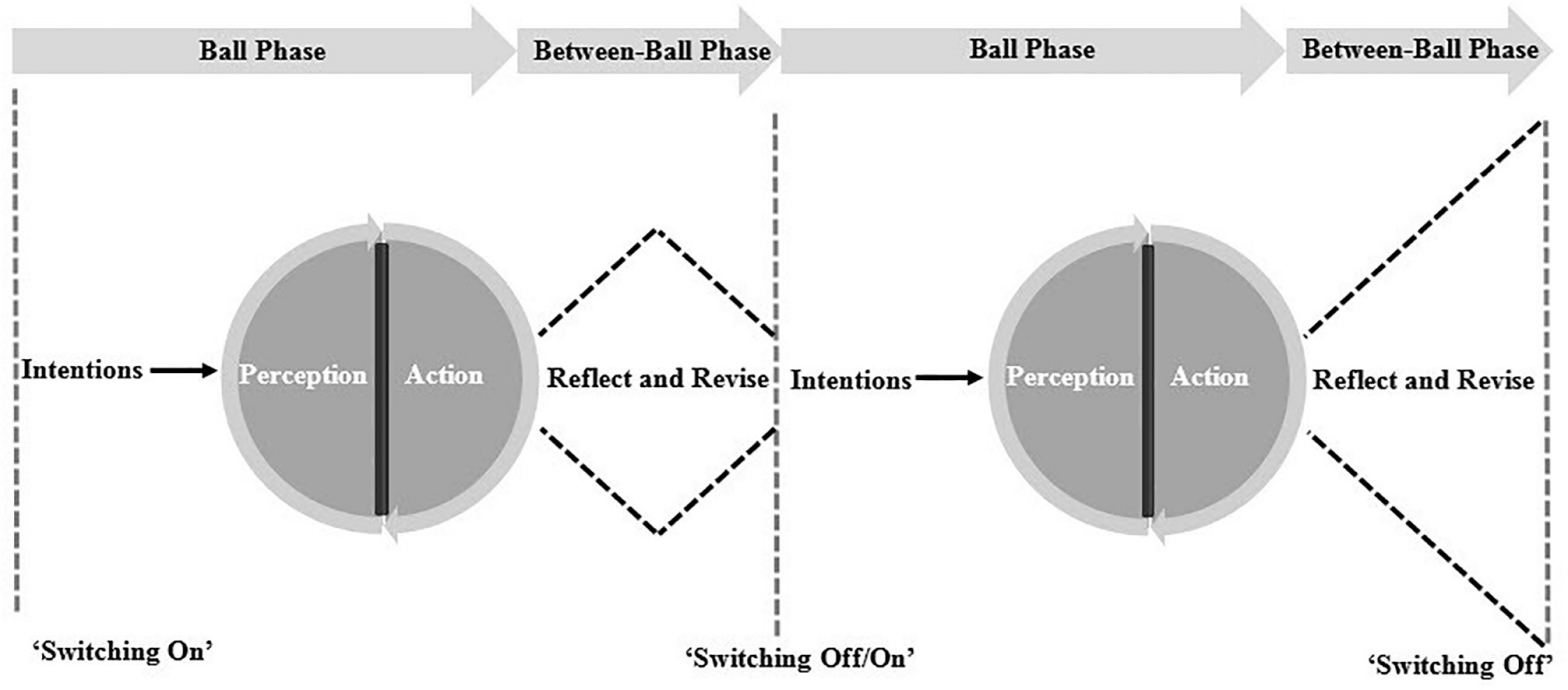
“The Plus” demonstrating the ball-by-ball cycle whereby expert batters engage in a reflective process, ‘switch off’ from task relevant thoughts, and ‘switch on’ again.

Another key finding of the study was that rather than simply batting being about each ball being treated as a separate event, expert batting can be described as a process of ball + ball + ball ^(n)^. Expert performance was significantly impacted by what batters did between balls, and thus, this phase has been coined “The Plus” (Fig 2). Given the dynamic nature of the batter-environment interaction during a batting event, expert batters engaged in a systematic between-ball process of reflection to update their ‘knowledge of’ the batter-environment system. This allowed them to update intentions, and more finely grained attunement to specifying information to exploit key affordances and (re-)calibrate actions. In this sense, cricket batting can be viewed as a continuous reflection of the key factors that relate to the ball, “plus”, all of the key factors that relate to the subsequent ball and the previous ball, “plus” all of the key factors that relate to the subsequent ball and the previous balls, and so on. This process enables batters to pro-actively manipulate individual, task or environmental constraints and allows them to meet their aforementioned goal to take control of the game.

### Pre-ball phase

#### Search for information ex situ (outside the performance environment)

Expert batter’s epistemological search for knowledge begins well before the actual batting innings (i.e. performance). During this early period, expert batters are concerned with collecting general information, funnelling down to more specific information as the time till the performance becomes closer. Gathering this information about the upcoming game allows them to begin to shape their intentions and formulate a plan against the opposition team.

*The art of batting*, *there are a number of things; reading the parameters*, *reading the conditions*, *this pitch*, *what are my shot making parameters on this pitch today*. *If you’re batting in Adelaide you’re probably driving at everything that pitches in your half*, *if you’re batting at the Gabba or the WACA*, *you’re only driving at half volleys and full tosses*. *So understanding those parameters was the first thing that was important*.

[IC IB^2^]

This funnelling of information concludes as the expert batter enters their performance environment (i.e. walks out to bat). While previous experience was noted as helpful, actually perceiving the affordances in the performance environments, and interpreting from this information what the opposition was likely to do, was the most reliable method to shape the expert batter’s intentions.

*Yeah you get out there*, *you take centre*, *you look around at your field*. *You’re not looking around to say g’day boys how you going*. *You’re working out okay I’ve got 4 slips and a gully*, *he’s not going to bowl too many at my pads… So you understand what they’re [opposition] trying to do*, *and that comes from playing against them as well*. *And if you play against someone you haven’t seen for a while or never seen them before–look at their field*. *Okay he’s going to try bowl outside off stump and swing it away*.

[SC IB^1^]

*Two*, *three days out really is when you should be really thinking about that [tactics and game plan]*. *[Another] quick go over [of your game plan] when you get to the ground*. *Have a think about who you’re facing*, *what they might do to you*, *how they usually bowl at you*. *But a lot of it*, *as an opener*, *especially if you haven’t played them before*, *is looking at the fields and summing up what they’re going to do when you’re out there*. *Having a really sold plan*, *or base game plan about what you’re going to do while your assessing that… It buys you some time to work out what they’re trying to do*, *and then evolve your game on top of that when you’re out in the middle*.

[SC IB^3^]

#### Understanding your own game

Framing intentions and formulating a plan prior to the game was crucial to expert batting performance. Experts shape their intentions around (1) their own action capabilities, (2) the affordances available in the performance environment, and (3) the task goal during a given situation of a game. The first crucial factor is that expert batters must possess superior knowledge of their own batting capabilities on various different pitch conditions in order to optimise their search for information.

*That comes down to understanding your game and making good decisions around that*. *So like if you are a bottom hand player*, *and exaggerate a strong bottom hand*, *you have to understand driving on the up on the onside is going to be quite tough for you*. *So if you get on a tough wicket you feel you can’t do that*, *you’ve got to have the decision-making and planning and discipline to say*, *right now I can’t do that today*, *or*, *I can’t do that for the first hour or 2; until the balls a bit older*, *or the wickets a bit flatter*, *or the ball is a bit closer to me*. *And then when the ball is under your nose you can do what you want with it then*. *But don’t go driving on the up to something slightly outside off stump swinging around if you’ve got a bottom hand grip [technique]*. *And that’s all apart of understanding your game*, *and putting it into you planning*.

[SC IB^2^]

One international coach described how four different international level cricket batsmen all devised different game plans to counter one opposition bowler. These intentions further highlight expert’s knowledge of their own superior coordinative actions and scoring options, they believe might be available to them, in the upcoming performance environment;

*I’ll give you an example… I think it was about 2004 and we went to Sri Lanka*, *and we hadn’t played [Sri Lankan Bowler] for a period of time… We set about*, *the coaching staff got divisioned together*, *got data together*, *then our first meeting in Sri Lanka we got the top six of the batting order together*. *And then we begin dissecting each other’s batting technique*. *And so [Aus Batter #1] for instance believed that his best method of playing [Sri Lankan Bowler] is that he would sweep as much as he could*. *So he had a big reach that*, *far bigger than anyone else’s*, *therefore that could upset [Sri Lankan Bowler]’s length for a start*, *aside for him the fact he played that shot very well*. *And if he couldn’t sweep he decided he’d get himself right back in the crease… [Aus Batter #2]*, *whose experiences in India were poor*, *said that if he played the way he played in India he’d be in trouble*. *So basically he said he was going to use his feet as much as possible and run at [Sri Lankan Bowler] as much as he could*. *[Aus Batter #3] said well even though he could use his feet*, *[he] said I can’t read him*. *So instead I’m just going to back myself and play deep*, *right in my crease and I’ll read him off the wicket–and I’ll back myself to do that*. *[Aus Batter #4] said first chance he got he would slog sweep [Sri Lankan Bowler] because he knows [Sri Lankan Bowler] didn’t like being hit so [Sri Lankan Bowler] would automatically move his field and that would open up some space where [Aus Batter #4] would be more comfortable playing that way*.

[IC SB^1^]

Secondly, the performance environment being cohabitated by the opposition and their own task goals meant the batters search for information for action, and the shaping of intentions, is a continuous, ongoing process. Expert batters described how their intentions were shaped by the affordances available to them. In this particular example, previous knowledge of an opposition bowler served to direct attention to search for certain opportunities for action.

*I knew with [former international fast bowler] he’s going to be running up bowling outswing*. *He’ll pitch most of them up and had a good bouncer*. *Before anything else I know that much*. *The next thing is*, *what happens when he makes a mistake*? *His mistake were going to be too full and too wide outside off stump so there cover drive*, *square drives straight drives*. *The other thing is if it doesn’t swing*, *it’ll slide onto my pads*, *so I’ve got the leg glance and the on-drive*. *So I know what my options are going to be before he has even bowled a ball*. *So now you don’t–you can’t go to the bank on that*, *because I don’t know what’s going to come out [of the bowler’s hand]*. *I’ve just got to make sure I’m ready and recognise straight away this is the full wide one–bang*. *This one is not swinging its sliding onto my toes- bang*! *You’ve got to have your game plan*

[IC IB^2^]

Finally, intentions were also shaped by the role of expert that would best serve the task goal. Cricket batting, being unique in that it has multiple different game formats that vary in duration, requires batsmen to balance the risk of a particular coordinative action (e.g. playing a shot in the air, or going for a quick run) and the subsequent reward (i.e. number of runs). This balance between risk and reward varies depending on the format of the game (e.g. limited-over game vs. multi-day game), the position of the game (e.g. number of runs scored/ required to win and the number of wicket lost), and the oppositions strategy (e.g. bowling plan and fielding positions). An international coach and batsmen described the role of an opening batsmen during a limited-overs game and how their task goals can reflect more risk-taking behaviour due to the constraints placed on opposition fielders (i.e. only a limited number of fielders can field on the boundary).

*[Regarding different batting roles at the beginning of a T20 game]… So you have an easy chance to clear the field and get the side off to a good start*. *Taking a few risks*, *but generally those players who bat up there*, *[it is] because they can play those shots*. *So [those shots are] not really risks–more playing their game*, *their role in the side if you like*. *But if it is nipping around a little too much*, *then they might have to reign it in a little*. *On the flip side if you go out and take him [the bowler] on a little bit*, *it might throw them off*. *In bowler friendly conditions they might start dishing up some poor balls*. *So there is a fine line*, *fine balance*. *That’s why those top skilful players can perform both those roles if you like*.

[IC IB^3^]

Importantly, coaches describe how the search for information about the upcoming performance environment subsequently shapes the intentions of the expert batsman. The information available also becomes increasingly specific as batsmen come closer to facing their first delivery. During this period, whether it be prior to the game or during the game, experts remain adaptable to changes that might influence their task goals (e.g. weather conditions, position of the game, etc.).

*… You have to be adaptable to change the momentum of the innings–whether that is by batting through an hour or whether it is counter attacking during a period*.

[SC IB^3^]

### Ball phase

When describing expert batsmen during this phase, it was highlighted that their actions are highly effective due to their attunement to the bowler’s kinematics. This inter-personal coupling to the opposition’s movements was suggested to allow experts to overcome extreme temporal constraints;

*He’s in time with the bowler*. *He’s got plenty of time*, *because he is picking up all of the information*. *You hear people say the difference between good players is they’ve got time*. *They’ve got time because they’re not handicapping themselves*. *They’re not making movements that are irrelevant*. *They’re pre-movements are in sync with the bowler*, *they’re picking up all the information so they do have more time than the guy who is not picking the ball up until its half way down*, *so of course he is rushed*.

[IC IB^2^]

While attuning to information is crucial, adapting one’s movements, based on newly formed intentions, was also an important component of expertise. Interestingly, this is accomplished to create an emergence and decay of possibilities for action.

*When we say watch the ball… You should be able to know the cues in watching the ball*. *It should almost be there seeing what they’re bowling before they even bowl the ball*. *So for me if they’ve got 3 slips and a gully*, *they’re not going to bowl short*. *If they’ve got back pad*, *leg gully*, *two back on the hook [shot]*, *they’re going to bowl short*. *So your pre-movements are not going to be forward*, *if they’re going to bowl short [you move back] so you give yourself more time*. *That’s watching the ball and understanding the game*.

[IC IB^1^]

It was further described that an efficient technique is achieved, firstly, by maintaining superior balance throughout the action and gripping the bat so that intuitive movements are less likely to result in a risky outcome (e.g. having a dominant bottom hand which can lead to the ball unintentionally being hit in the air). Secondly, coaches suggested that expert batters are less inclined to make superfluous or unnecessary movements. Interestingly, comments on technique were often intertwined within the context of intentionality (i.e. take control of the game, clear mind);

*I’d say the key thing is good footwork*, *clear mind*. *I think your head position–keeping your eyes horizontal are key to picking up line and length well*. *Tempo or whatever*, *footwork*. *I mean you can go on more the grip*, *the bat-lift and all sorts of things*

[SC IB^2^]

When asked about tempo, this coach referred to the concept of “having plenty of time” [follow up question–“What did you mean by tempo”?].

*Really decisive footwork is how to put it*. *If you’re going forward then your [going] forward*, *if you’re going back than you’re going back*. *There’s no stuck in the crease or in between and no second guessing yourself*, *your first decision is the right decision*.

[IC IB^1^]

Interestingly, the initial phase of the expert’s batting innings is highlighted as the most difficult. In order take advantage of the affordances presented within the environment, batters are required to attune to specifying sources of information (e.g. pitch, opposition bowler and strategy) and adapt their coordinative actions to suit;

*What we talk about as coaches*, *we talk about our first 20 or 30 balls… Hit the ball here*, *do this foot work*, *and your back lift is here*. *And some people misconstrue this*, *and think*, *well that’s how I’ve got to bat for the whole innings*. *Nah*, *that’s how you bat until you get a feel of what’s going on in the middle…*. *Once you get in*, *you can actually start playing the shots you want cause you understand what the wickets doing*, *you understand what the balls doing*, *you understand what the bowlers trying to do*.

[SC IB^2^]

*So [at the start of your innings] you’ve generally got a period of about half an hour where it might be tough*. *Another time where it’s overcast*, *you might have to work for 20 overs before it becomes simple or before you feel like you’re in control of the game… So generally when there is conditions like this [a flat pitch that provides consistent ball trajectory and little to no lateral movement (swing)]*, *you feel like you can actually hit the ball freer*, *you see it better and you can hit it*. *In conditions which favour the bowler*, *the ball moves*, *which tests your technique*. *So anything that moves is dangerous*, *and anything that doesn’t move is fodder*.

[SC IB^4^]

Coaches describe that a batter’s primary goal is being able to ‘control the game’. This task is accomplished by assessing the position of the game, and taking advantage of available scoring opportunities congruent with the amount of risk involved taking them.

*…you’d say that world best are generally making good decisions on bowlers or on state of game or on the conditions*. *And then tied into that would be their tactical nuance*. *With the state of the game*, *what do they do*? *How do they work out how to score runs*? *How do they try to put themselves in a position where they control the game and the bowler*?

[IC SB^1^]

*Developing your game where your routine [development of game plan] is the same on every surface*, *to start with*. *Then from that routine you work out*, *okay*, *these are the shots I can play today*. *These are the types of bowler’s I’m facing*. *This is where I’m looking to score my runs*. *Battings not about survival*, *it’s about scoring runs* … *If you have the same routine*, *same… philosophy at the start [of your innings]*, *you can then gauge what you can play and what you can’t play*.

[SC IB^1^]

#### Intentions shaping perception-action

During performance, experts must reshape their intentions, actions and perception continually to adapt to the situation of the game, underpinned by their overarching goal of being ‘in control’. In regards to intentionality, experts are better able to recognise key nested events that offer opportunities to manipulate the opposition, by executing certain actions themselves;

*I don’t know whether you’ve heard me talk about the danger zone*, *that 4 to 6 metre mark*, *that’s the danger zone*. *If the bowler owns that*, *then he’s on top*. *You [the batter] own that*, *and you’re on top*. *The job of the batsman is to get control of that danger zone as quickly as possible*. *So if he slides a bit full to my side of the danger zone*, *I have to punish him*. *If I punish him*, *if I hit him back down the ground for four*, *I can guarantee you that apart from the top 1% of bowlers*, *the next ball will not be up there*. *They’ll adjust and they’ll try and bowl back of the length*, *which often comes short*. *And bang*, *the next one goes for four and guess who’s under the pump*?

[IC IB^2^]

Coordinative actions are, similarly, reshaped to suit the demands of the performance environment. These adaptations to actions may only be temporary, until certain constraints evolve and present new opportunities for action. Changes in constraints, which lead to novel affordances, may occur through a change in fielding positions, opposition bowler, or a change in ball flight characteristics (i.e. less lateral movement).

*This only came late in my career but I visualised batting in a phone box*. *And I wasn’t allowed hit the ball until it was right into my phone box*. *So say you’re batting in a phone box*, *you can’t hit the ball till it arrives there and it stops you from hitting out here [in front of your body]*. *And this is once again misconstrued information–it’s not for your whole innings*. *Some days it might be*, *because the bowler’s all over you*, *and the balls swinging and doing all sorts*. *And if the ball’s swinging and you’re playing out here [in front of your body] you’re in [expletive] trouble*.

[SC IB^1^]

Experts also modify their perceptions as the situation of the game changes. Affordances that were previously exploited, may no longer equal the risk-reward balance experts perceive. Depending on the change in constraint (e.g. loss of wicket might require more conservative behaviour), experts modify their search for affordances that present the lowest degree of risk that would still achieve the task goal. This particular coach describes some examples whereby an expert might look for a specific scoring area to exploit, or modify their intentions towards each opposition bowler they face;

*If you can see a batter who is going really well*, *and a couple of wickets fall*, *is able to reign himself in and build another partnership*. *And then he might go through the gears again*. *In a one-day game*, *it’s being able to pick [and] attack the bowlers you feel confident hitting*, *or what areas you feel you know you’re going to hit best*, *and then judging an innings*. *If you’ve got to chase 300 these days*, *there has got to be some good hitting in there*, *and have a good plan on who you may target*, *what areas of the field are your areas*, *are your zones*. *That’s just batsmanship*. *Or craftsmanship*. *That’s just understanding what you’ve got to do*.

[SC IB^1^]

Finally, manipulating and exploiting affordances in the performance environment is an ongoing and continual process during a game. Two different expert batsmen can manipulate and exploit the environment in vastly different ways. While these batsmen may share the same goal, their perception on the environment, relative to their own capabilities may differ, and thus, will shape their intentions and actions differently. Coaches described how having different intentionality-style batsmen go about maintaining a sense of control over the game, through shifting perceived pressure onto the opposition, can be more effective;

*Pressure [back onto the bowler] is built in different ways*. *Whether that’s taking the game deeper into the innings by taking shine off the ball*, *and minimising damage [i*.*e*. *losing wicket] with the top order… or by scoring runs at a good rate*. *I think if you’ve got two guys trying to take the shine off the ball*, *the pressure doesn’t build on the bowler at any stage*, *then if all of a sudden you lose a couple of wickets*, *the team is under pressure*. *But if you’ve got a guy taking the shine off the ball and another scoring runs than that’s a good mix*.

[SC IB^3^]

When asked whether expert batters could interchange between different roles, the same coach further described how this intentionality-style role is often reflected by your understanding of your capabilities;

*There are definitely players who are adaptable and can do both*, *but generally speaking its one side of the fence or the other…*. *I always tried to have an aggressive mindset with the way I played but I was limited in my abilities in certain areas*, *areas I just didn’t touch until 2 hours into my innings*. *And then I was able to go from there and try take the game away*.

[SC IB^3^]

### Between-ball phase

In-between deliveries were highlighted as crucial periods for expert batsmen. Constant evaluation of the performance environment, especially over periods of multiple days, required certain strategies to manage certain stressors. The follow section explores the purpose and processes of a between-ball routine exhibited by expert batsmen;

*The purpose of a good routine is to prepare yourself properly*. *To make sure that you are in the right frame of mind to receive the next delivery*.

[IC IB^2^]

*A very important part of batting is how you*, *what you do between balls*. *What you think about*, *and how you let the previous [ball] go*, *and then prepare [yourself] to be ready for the next one*. *The art of batting is very much; the physical aspect of it is the part we see*, *but that’s the tip of the iceberg*.

[IC IB^2^]

Following a between-ball routine suggestibly helped experts, when in their performance environment, to regulate their emotions. Coaches described how experts reconciled their thoughts to ensure they felt a sense of comfort, and, similar to the shaping of their intentions, take control of the game situation.

*[Talking about having a routine to follow] But it’s for us to know we can walk out there and be in that feel good comfortable space*. … *And the mental side [of a routine] is [utilised] for feeling confident*

[SC IB^4^]

*Oh you’ve got to be [comfortable]*. *I had a few minutes with [former international batter] chatting and he said*, *he felt the most important thing he felt is you had to be comfortable in your environment*. *Comfortable in the middle–to him that was his territory*. *The swagger that was his*, *[he] had to get comfortable out there and dictate*, *control and make sure you know you’re the boss of things*.

[SC IB^2^]

Between-ball routines also provided an opportunity for batsmen to reflect on the current situation of the game, and the accompanying nested events that occur throughout. This cognitive reflection can range from the previous deliveries, to the next perceived event. Taking advantage of immediate affordances was described as a crucial component of maintaining control of the game. However, forethought to next period of the game (i.e. nested event) was also a part of evaluating current demands, and therefore, intentions;

*It starts ball by ball*, *if you’re managing yourself well and you’re playing each ball on its merits then you’re at least even*. *Then it’s a matter of are you taking all of the scoring opportunities*? *And provided you’re taking most of them you’re on top… He knows if I’ve missed a scoring opportunity*. *He’s bowled a bad ball or less than good ball and you haven’t scored of it*, *he knows he’s dodged a bullet*. *And if he keeps bowling them and you keep not scoring off them then he’s under no pressure*.

[IC IB^2^]

*What’s your strategy*, *if you went another five overs deeper would it [the game situation] change*? *Would they have brought on a different bowler*? *Or would they have been a little more tired*? *Would you get more loose balls if there further into their spell*? … *Try get them to see hey if I can wrestle [through] this period or hey they [the opposition] might go away from their plan too if they haven’t got you out*. *[international bowler] is on for a reason–he’s on to try get you out*. *If he’s not getting wickets*, *what’s he going to do*? *He’s going to change his plan*. *Is that plan going to be more suited to your game when they’re bowling bouncers at you*? *Most likely*. *Maybe not*. *But it’s going to be different from not being able to score right now*. *So being able to understand that*.

[SC IB^3^]

However, it was also highlighted that, while it is important to reflect on errors, it is just as important not to dwell too long upon them;

*… So as cricketers we miss them all the time [taking advantage of a perceived scoring shot]*, *and you have to just reset and refocus*.

[SC IB^4^]

The physical component of the routine was described as a manifestation of taking a break from the demands of the performance environment. Various actions were employed, such as walking down the pitch and tapping the ground (i.e. gardening), talking to a teammate or looking into the crowd. As such, task irrelevant thoughts were very common during this switch off period;

*I was pretty calm out in the middle*, *not much fazed me out in the middle*. *I liked to score*, *so when I wasn’t scoring I could get a bit itchy*. *Especially the younger version of me* … *I remember looking at the score*, *or float around [looking at] the crowd*, *or wander down the wicket [and] say something to my mate*, *a bit of gardening… Always quite consistent*, *what I did*.

[IC IB^3^]

*… Everybody has a routine*. *When I talk to people*, *particularly good player’s*, *their routines aren’t that dissimilar*. *There is a physical aspect to it*, *at the end of each ball they have a break so they might walk down the pitch and pat down imaginary things*, *or they might walk out towards square leg just take a few steps away and walk back in again*. *It might involve marking their guard either every ball or it might be they just mark their guard again when they’re back on strike or at the start of a new over*.

[IC IB^2^]

This ‘switch off’ period in-between deliveries was highlighted as crucial for expert batsmen. This strategy was suggested to assist in overcoming any mental or cognitive fatigue that might occur during performances that stretch for hours or across days.

*I liken it to a motor car; you get in and you turn the motor car on*. *The cars in neutral–so the engine’s ticking over but it’s not using a lot of gas*. *That’s between balls*, *that’s between overs*, *that’s waiting to go into bat*. *The engine’s on*, *you’re aware of what’s going on*. *Then general awareness*, *but you’re not using up a lot of energy… So when the bowler gets back to the top of his mark*, *you put it into first gear*. *So now you’re ready to roll*. *But again you’re not using up a lot of energy… And as the bowler gets into the load up*, *you stick the car into over drive*. *Because now*, *from that point to the time you receive the ball*, *which is probably less than a second*, *is the critical moment… So from then until the play was completed I was in overdrive*, *and that would have only have been a few seconds*. *Once the play was dead*, *I put the car back into neutral*.

[IC IB^2^]

*Well the contest starts basically just as the bowler starts running in*, *so if you can be in that moment of contest and switch on for however long you’re going to bat for*, *[then] that’s your job*. *That’s all you have to do*. *You have to be engaged for that moment*.

[SC IB^4^]

## Discussion

The purpose of this study was to better understand cricket batting expertise from the perspective of individuals with knowledge of expertise originating from both coaching and playing at an elite level. The findings of this study further expand on Weissensteiner, Abernethy [30] initial conceptual model of expertise development in cricket batting by developing a temporal model of performance expertise. The themes that emerged included exploring how an expert’s perceptual skills and attunement to the environment shapes their individual technical (motor) skills; while their cognitive strategies manage their decision-making through self-regulatory behaviours, and psychological stresses. Coaches described expertise as a multi-faceted, co-adaptive relationship between the individual and the environment to gain a perceived ‘control of the game’. The changing environment included the opposition bowler and their tactics, pitch conditions and the situation of the game. The model reveals that the expert batter needs to be attuned to this information, as they shape the intentions of the individual’s actions (that is, technical motor skills) relative to the affordances within the environment.

The strategy by which an expert cricket batter changes from one previously-functional movement to another now-more-functional movement, based on changes occurring in the environment, can be better understood using behavioural dynamics [38]. Experts are regarded as possessing superior technical skills that can be considered stable behavioural patterns (i.e. actions), consistently reproducible and resistant to certain perturbations. However, they also demonstrate flexibility within their movements that allows for adaption; tailored to the performance environment. Bifurcation is the mechanism by which one behavioural pattern is no longer considered functional, and instead the expert batter adapts towards another behavioural pattern [39]. This switch between stable patterns, as described by Araújo, Davids [40], is considered as a result of the changes in the environment. Similarly, expert batters suggestibly adjust previously functional techniques (e.g. footwork) and adapt to the changing conditions brought about by the opposition bowler and affordances within the environment.

Coaches regularly described scenarios where experts could utilise relevant and available information in their performance environment to shape their intentions, and as such, their actions. Self-regulatory behaviours, such as the planning, monitoring and evaluating actions, are suggested to explain how expert batters manage and manipulate the constant changes occurring in the performance environment. While self-regulation research itself is still developing outside of academia, it is relevant to note that it is the environment which stimulates an individual’s awareness, and subsequent regulation [41]. Without this interaction of the individual and environment, the act of self-regulation does not occur. More broadly, Zimmerman [42] surmised the concept as *“the degree to which students are meta-cognitively*, *motivationally and behaviourally active participants in their own learning process”*. In this instance, it is suggested that every performance can be likened to a batter being required to ‘learn’ what is required to succeed. The process of how expert batters go about achieving this is presented in Fig 1.

Developing a routine to manage stressors, both externally and internally produced, was another characteristic of expertise. Internal stressors included mental fatigue and lapses in concentration, while external are those that were exacerbated by environmental constraints such as maintaining a level of comfort and control within the performance environment. Expert batters employ routines, both behavioural and cognitive, in between deliveries to manage these pressures. Similar to the research findings on performance routines [43, 44], this suggestibly allows experts to manage more effectively their emotions; attentional focus [45, 46]; concentration [47] and enhance consistency of performance [48, 49]. Interestingly, a crucial aspect of the pre-performance routine literature is said to involve being able to channel attention from irrelevant thoughts to task-specific thoughts. In contrast, coaches described using irrelevant thoughts between deliveries as an effective strategy to prolong optimal arousal states. One explanation for these conflicting approaches may be that research on pre-performance routines has predominately focused on more closed skills (i.e. those with specific start and end points). Therefore, tasks that involve on going, *in-performance* routines, that occur over extremely long periods of time (in the example of Test cricket, a 5 day game involving 3 hour playing sessions before a break, 3 times per playing day) may require alternative strategies.

The final key finding was an expert’s knowledge of the performance environment; that is knowing what is required and how to achieve it based on own perceived strengths. Experts were described as being able to assess conditions and recognise which of their own repertoire of coordination patterns yield the lowest risk for the most reward, depending on the situation of the game. Having effective coordinative and tactical strategies when the trajectory of the delivery promotes meta-stability. Pinder, Davids [50] provided evidence for a meta-stable performance region in cricket batters when the ball is pitched between 4m and 8m, demonstrating a mix of front and back foot, and attacking and defensive coordination patterns. Interestingly, a coach (international level coach and player) highlighted how this area is crucial for a batter to maintain a sense of control over. The purpose of this is that deliveries pitched closer to the batter demonstrate a more stable movement pattern, similar to balls pitched further away, and therefore easier scoring opportunities. When pitched on this ‘good length’ (4m–8m from the stumps; common term referred to by coaches and players), expert batters are suggested to have ‘intentions’ (e.g. game plan) that help weight the execution of certain coordination patterns over others. These intentions are formed from the individual’s constraints (e.g. emotions, cognitions) and available information in the performance environment.

Expertise has often been explored as a ‘snapshot of the performer at a single point, or over a very short period of time [26]. However, it is becoming more common for researchers to exploit alternative methodologies that allow for a more holistic understanding. Gaining the perspective of former expert batters, who then became elite level batting coaches, provides a unique perspective on expertise. In this instance, expertise is not expressed as a snapshot during a performance; instead, as a model of how it is repeatedly characterised over multiple performances. Future work could address some limitations within this study design, such as experimentally examining the importance of the various components of expertise identified in the study. While some limitations are unavoidable with this methodological approach, for example potential participant or researcher bias, interviewing expert coaches can provide unique insights on expertise that are not easily accounted for in controlled environments. Greenwood, Davids and Renshaw [28] reinforced that, while the utilisation of coaching expertise empirically is under-represented, it can complement existing evidence and provide avenues for future direction.

## Conclusion

The findings from this study provide support for viewing expertise as multi-dimensional. Cricket batting is one such example where the technical, tactical, perceptual and psychological skills interact to underpin expert performance. Expert batters ‘control the game’ by perceiving the changing affordances in the performance environment; that is, assessing whether the performance environment favours the expert batter, and then exploiting certain bowlers or periods of time until it does so. Through an awareness of their technical strengths and perceiving the game situation, they are able to minimize the risk of being dismissed while shifting pressure back onto the opposition by scoring runs. Finally, batters possess well developed psychological strategies to manage emotions such as anxiety, and problem solve game specific challenges. Future research should endeavour to investigate individual differences between experts, and how to effectively develop batting expertise.

## Supporting information

S1 Fig (JPG)
