# Supplementary figures and images for: Defining cricket batting expertise from the perspective of elite coaches
Source: PLoS One. 2020 Jun 15;15(6):e0234802. doi: 10.1371/journal.pone.0234802 (PMC7295192; doi:10.1371/journal.pone.0234802)

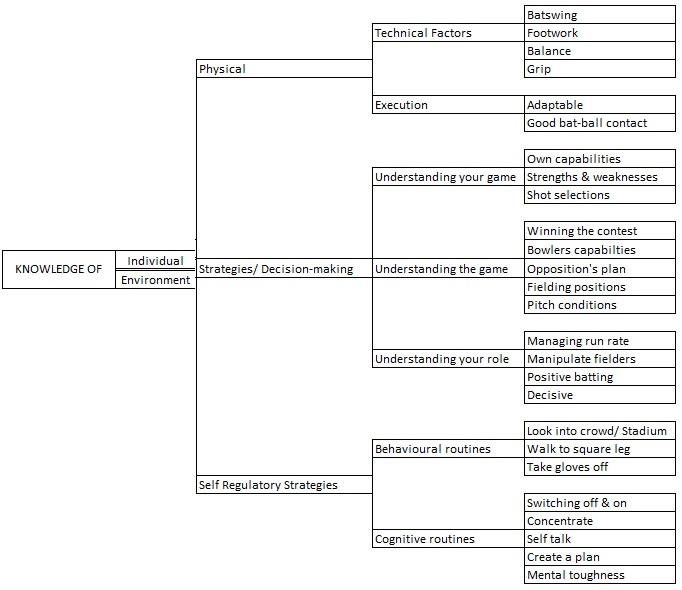

Supplement: S1 Fig — (JPG) [file pone.0234802.s001.jpg]
